# Determinants of Amyloid Formation for the Yeast Termination Factor Nab3

**DOI:** 10.1371/journal.pone.0150865

**Published:** 2016-03-08

**Authors:** Thomas W. O’Rourke, Daniel Reines

**Affiliations:** Department of Biochemistry, Emory University School of Medicine, Atlanta, Georgia, United States of America; Ruhr University Bochum, GERMANY

## Abstract

Low complexity protein sequences are often intrinsically unstructured and many have the potential to polymerize into amyloid aggregates including filaments and hydrogels. RNA-binding proteins are unusually enriched in such sequences raising the question as to what function these domains serve in RNA metabolism. One such yeast protein, Nab3, is an 802 amino acid termination factor that contains an RNA recognition motif and a glutamine/proline rich domain adjacent to a region with structural similarity to a human hnRNP. A portion of the C-terminal glutamine/proline-rich domain assembles into filaments that organize into a hydrogel. Here we analyze the determinants of filament formation of the isolated low complexity domain as well as examine the polymerization properties of full-length Nab3. We found that the C-terminal region with structural homology to hnRNP-C is not required for assembly, nor is an adjacent stretch of 16 glutamines. However, reducing the overall glutamine composition of this 134-amino acid segment from 32% to 14% destroys its polymerization ability. Importantly, full-length wildtype Nab3 also formed filaments with a characteristic cross-β structure which was dependent upon the glutamine/proline-rich region. When full length Nab3 with reduced glutamine content in its low complexity domain was exchanged for wildtype Nab3, cells were not viable. This suggests that polymerization of Nab3 is normally required for its function. In an extension of this idea, we show that the low complexity domain of another yeast termination factor, Pcf11, polymerizes into amyloid fibers and a hydrogel. These findings suggest that, like many other RNA binding proteins, termination factors share a common biophysical trait that may be important for their function.

## Introduction

The study of low complexity protein domains (LCDs), particularly those that are considered intrinsically disordered, has gained focus recently because of the unusually large number of proteins that contain them, and their association with prion-like structural properties [1, 2]. RNA binding domains are over-represented in proteins with prion-like regions. While 1% of the proteins in the human genome have an RNA recognition motif, ≈12% of proteins with a prion-like domain have an RNA recognition motif [2]. LCD sequences commonly lack stable, folded secondary structural elements and have been referred to as intrinsically disordered [1]. Although unstructured, these domains can assume stable folded states through intermolecular aggregation or polymerization; in some cases assembling into regular filaments which can further organize into hydrogels [3]. Some of the proteins bearing these domains form heritable prion-like particles [4]. A bioinformatics analysis of the yeast proteome revealed that many recombinant low complexity prion-like domains formed amyloid polymers *in vitro* [5]. *In vivo*, some of the candidates displayed features of aggregation and some were able to stimulate prion formation. This initial survey has been seminal in the study of LCDs. We recently showed that LCDs from the transcription termination factors Nrd1 and Nab3 which were predicted to be prion-like, indeed form amyloid-like filamentous polymers *in vitro* [6].

One mechanism for terminating transcription by RNA polymerase II (pol II) employs the hnRNP-like proteins Nab3 and Nrd1 and the helicase-like protein, Sen1 [7]. The Nab3-Nrd1-Sen1 (NNS) complex is the major termination machinery for short, non-coding RNAs. Nab3 and Nrd1 bind RNA through a conserved RNA recognition motif [8, 9]. Sen1 is thought to unwind the nascent RNA from the transcription bubble [8]. The three proteins interact with each other, with pol II, and with nascent RNA. They also recruit the TRAMP complex and nuclear exosome to process or degrade RNA substrates [10, 11]. This complex network of interactions is important for termination activity as pairwise mutations that disrupt these interactions are deleterious and often lethal [12]. Nrd1 has a motif (CTD-interaction domain [13], CID) that has been shown to bind the carboxy-terminal domain (CTD) of the pol II’s largest subunit [14, 15]. Interestingly, other proteins that associate with pol II, including subunits of the Mediator complex (Med2, Med3, Med15) and termination factors associated with cleavage and polyadenylation of mRNA (Pcf11, Hrp1, Rat1), also contain prion-like domains as predicted by a Hidden Markov Model algorithm [5]. Med3, Med15, and Hrp1 have been shown to form amyloid assemblies [5, 16]. The termination factor Pcf11, like Nrd1, contains a CID that binds a specific phosphorylated form of the pol II CTD [17, 18]. Curiously, the mammalian RNA-binding protein FUS, which has an LCD and forms protein polymers, has been shown to interact with the pol II CTD in a phosphorylation-dependent manner, suggesting that this may be an additional type of contact that enables the recognition of pol II across the transcription cycle [19].

Recently, it has been shown that Nab3 can employ its LCD to detoxify a derivative of the polyglutamine-containing huntingtin protein in living yeast [20, 21]. This is thought to operate through a direct interaction of the two polyglutamine-containing proteins. Prior work showed that a portion of the Nab3 LCD fused to GFP formed punctate aggregates in yeast as observed by fluorescent microscopy [5]. These findings suggest that Nab3’s polyglutamine domain can be functionally deployed *in vivo* to form higher order structural assemblies. Nab3 contains a lengthy prion-like domain that is glutamine and proline rich over 235 amino acids, including a tract of 16 glutamines. The domain ends with a C-terminal region with structural homology to an α-helix found in human hnRNP-C that is known to self-associate into bundles [22]. Mutations in this part of Nab3 were picked up as terminator override mutants in yeast [23]. Loss of the final 134 residues, is lethal [12]. Genetic and biochemical evidence suggest that multiple copies of Nab3-Nrd1 dimers bind to nascent RNA during the termination of transcription [9, 12]. Recent work on a number of RNA binding proteins also suggests that higher order LCD interactions are important for compartmentalization of RNA metabolism [24–27]. Hence, a working hypothesis is that the LCDs of RNA-binding proteins enable non-membrane delimited compartments to form that house RNA metabolism centers [3]. Here we have investigated the determinants of filament formation of Nab3’s low complexity domain. Recently, the expression and purification of full-length Nab3 has been reported [28]. We now show that, as observed for its isolated LCD, the entire protein also forms amyloid fibers. As well, the termination factor Pcf11’s low complexity domain, forms filaments and a hydrogel. These findings suggest that self-assembly of low complexity domains is an important part of the function of proteins that interact with pol II and in particular, factors involved in terminating transcription.

## Materials and Methods

### Plasmid and protein preparation

Full-length Nab3 expressed from a modified pET41a plasmid (pDL469, courtesy of D. Libri) was transformed into BL21(DE3) *E*. *coli* and expressed by autoinduction as described [28]. Cells were resuspended in lysis buffer (50mM Tris, pH 7.5, 500mM NaCl, 10mM imidazole) containing protease inhibitors (EasyPack, Roche, Inc.), lysed with 10 μg/ml lysozyme, sonicated, and centrifuged at 27,000 X g for 30 min. Supernatants were applied to a 1 ml HisTrap HP nickel column (GE Healthcare) equilibrated in lysis buffer, washed with 25 ml of lysis buffer, and eluted with lysis buffer containing 250 mM Imidazole. Fractions were pooled and digested with TEV protease, for 16 h at 22°C. Digested proteins were exchanged into lysis buffer by centrifugal filtration in Vivaspin units (GE Healthcare) and re-run on a HisTrap HP column. Nab3 in the flow-through were concentrated with Vivaspin units with intermittent pipetting to promote mixing while exchanging into 0.2M NaCl, 50mM Tris, pH 7.5. Amyloid formation was initiated by incubating concentrated, purified protein, at 4 degrees without agitation. Viscosity and gel formation were monitored visually. At varying times following incubation, extending from days to weeks, samples were taken for amyloid and filament formation assays. While not quantified, filament formation tended to proceed faster at higher protein concentrations and thus varied from preparation to preparation and protein to protein.

The sequence of Nab3 encoding its C-terminal domain was deleted from pDL469 using Phusion DNA polymerase (NEB) and the oligonucleotides 5’-aggtggttgaggaggcggacc-3’ and 5’-gaaaacctgtattttcagggacaccacc-3’. This plasmid (pET41-Nab3Δ134) was transformed into BL21(DE3) cells and this truncated version of Nab3 was expressed and purified as described above for full-length Nab3.

**Fig 1:**
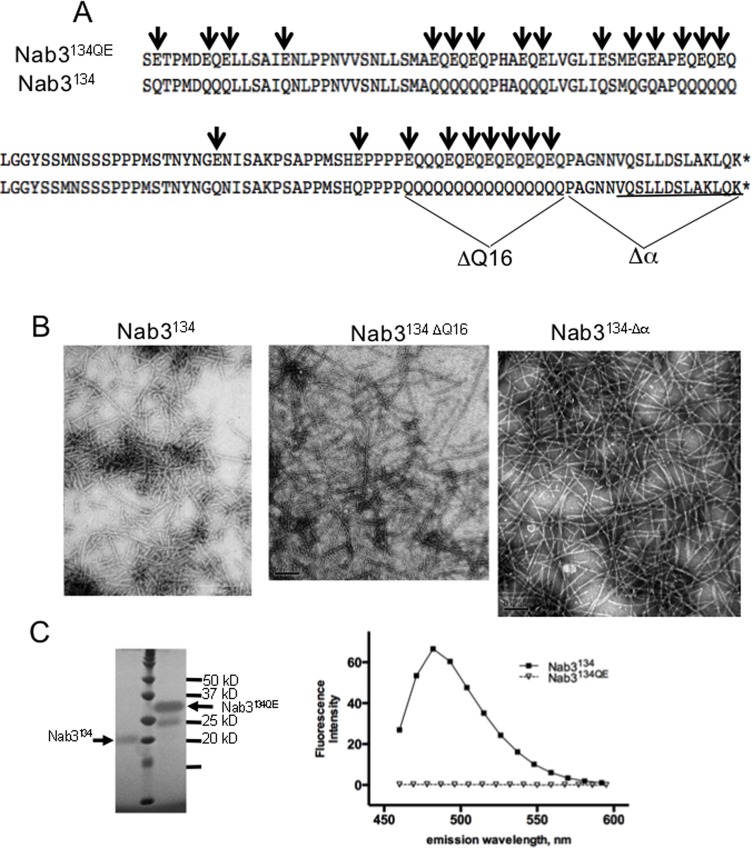
Filament formation by mutants of the LCD of Nab3. (A) A portion of the wildtype (Nab3^134^) and glutamine to glutamate substituted (Nab3^134QE^) LCD of *S*. *cerevisiae* Nab3 is shown. The extent of the deletion of Q_16_ run and the terminal hnRNP-C-like domain (Δα) are also shown. The region of Nab3 with structural homology to human hnRNP-C is underlined. (B) Transmission electron microscopy images of filaments from the various purified versions of Nab3^134^ are shown. (C) Purified Nab3^134^ and Nab3^134QE^ were run on a 12% SDS-PAGE gel (left panel) next to molecular weight markers and stained with Imperial^TM^ stain (note this polypeptide binds Coomassie blue-based dyes poorly). Thioflavin T-binding and fluorescence (right panel) was measured for Nab3^134^ and Nab3^134QE^ as described in Materials and Methods.

Derivatives of the Nab3 low complexity region (Nab3^134^) protein were expressed from plasmids pET32a-Nab3-134, pET32-Nab3^134ΔQ16^, pET32-Nab3^134Δα^, and pET32-134QE. pET32a-Nab3-134 has been described [12], it encodes the C-terminal 134 amino acids of Nab3 fused to thioredoxin by inserting the coding sequence between the *Bgl*II and *Xho*I sites of pET32a. pET32-Nab3^134ΔQ16^, was made by deleting the DNA encoding the stretch of sixteen Qs from pET32a-Nab3-134 using Phusion DNA polymerase and the oligonucleotides 5’-cctgctggcaataatgttcaaagtctatta-3’ and 5’-aggtggcggaggttggtgtgac-3’ in mutagenic PCR. pET32-Nab3^134Δα^ was similarly made by deleting the last 18 amino acids of Nab3 from pET32a-Nab3-134 using the oligonucleotides 5’-aggttgctgttgctgttgctgttgctg-3’ and 5’-tgactcgagcaccaccaccaccac-3’. pET32-134QE was created by synthesis (Genscript, Inc.) of the portion of Nab3 shown in Fig 1A in which 24 Q codons were changed to glutamate codons inserted into the *Eco*RV site of pUC57. Synthesis included *Bgl*II and *Xho*I sites at the 5’ and 3’ ends respectively which were used to excise the fragment for insertion into similarly digested pET32a. All plasmids were transformed into *E*. *coli*, which were induced with IPTG, lysed with lysozyme, sonicated, and the lysate was chromatographed over HisTrap media as described above. Pooled fractions were digested with thrombin, washed back into lysis buffer, and re-chromatographed on HisTrap columns as described above. The resulting proteins were concentrated by centrifugal filtration and left undisturbed at 4°C or 22°C.

pET32-Pcf11-LCD was assembled by synthesizing the sequence from codon 228 to codon 287 of Pcf11 with *Bgl*II and *Xho*I sites on its ends for insertion into similarly cut pET32a. The plasmid was transformed into *E*. *coli* for expression and purification of the thioredoxin-Pcf11 LCD fusion protein. Cells were lysed and chromatographed on HisTrap as described above. Eluted protein was cleaved with thrombin and re-chromatographed on HisTrap as described for the Nab3 polypeptides.

Plasmids for introduction into yeast started with pRS315-Nab3 [12]. DNA encoding Nab3 amino acids P^609^-K^802^ with 24 glutamine to glutamate substitutions (Fig 1) encompassed on an *Nde*I-*Apa*I fragment, was synthesized (Genscript, Inc.) and inserted in place of the corresponding wildtype fragment in pRS315-Nab3 to create pRS315-Nab3^24Q>E^. Control plasmids containing 11 or 14 glutamine to glutamate substitutions in this region were made through multiple rounds of site-directed mutagenesis using mutagenic oligonucleotides to generate the plasmids pRS315-Nab3-11QE and pRS315-Nab3-14QE. The plasmid pRS315-*PCF11* [23] was used to delete its core prion-like domain (residues 228–287; as defined by Alberti *et al*., [5]) by mutagenic PCR using the oligonucleotides 5’-ttatcttcctctctttttggtaatatttctggcg-3’ and 5’-tctttcctgcaaaacttgttggtcctg-3’ and Phusion DNA polymerase. The resulting plasmid was pRS315-*PCF11-ΔPrLD*.

### Yeast Strains

Strains used in this study are listed in Table 1. The strain DY3111, bearing a chromosomal deletion of *NAB3* and a wildtype copy of *NAB3* on a *URA3*-marked plasmid, was used as a starting strain for plasmid shuffling. It was transformed with the LEU2-marked plasmids pRS315-Nab3-11QE, pRS315-Nab3-14QE, or pRS315-Nab3 to yield strains with two plasmids. Counterselection against the *URA3* plasmid on 5-fluoroortic acid (FOA)-containing medium yielded the strains DY368, DY384, and DY357, respectively. DY382 was made by transforming DY3111 with pRS315-Nab3^24Q>E^. For *PCF11* shuffles, H-314 (courtesy of Dr. M. Hampsey) was used as a starting strain. pRS315-*PCF11* or pRS315-*PCF11-ΔPrLD* were transformed into H-314 to generate DY361 and DY362, respectively. Counterselection against the *URA3*-marked plasmid following growth in the presence of FOA led to strains DY363 and DY364, respectively.

**Table 1:** 

DY357	MATalpha *ura3Δ0 his3Δ1 leu2Δ0 nab3Δ0*::*kanMX* [pRS315-NAB3 (*LEU2*)]	This study
DY361	MATa *ura3-1 trp1Δ ade2-1 leu2-3*,*112 his3-11*,*15 pcf11Δ*::*TRP1* [pFL38-*PCF11* (*URA3*)] [pRS315-*PCF11* (*LEU2*)]	This study
DY362	MATa *ura3-1 trp1Δ ade2-1 leu2-3*,*112 his3-11*,*15 pcf11Δ*::*TRP1* [pFL38-*PCF11* (*URA3*)] [pRS315-*pcf11ΔPrLD* (*LEU2*)]	This study
DY363	MATa *ura3-1 trp1Δ ade2-1 leu2-3*,*112 his3-11*,*15 pcf11Δ*::*TRP1* [pRS315-*PCF11* (*LEU2*)]	This study
DY364	MATa *ura3-1 trp1Δ ade2-1 leu2-3*,*112 his3-11*,*15 pcf11Δ*::*TRP1* [pRS315-*pcf11ΔPrLD* (*LEU2*)]	This study
DY368	MATalpha *ura3Δ0 his3Δ1 leu2Δ0 (lys2 and met15 status unknown) nab3Δ0*::*kanMX* [pRS315-Nab3 11QE (LEU2)]	This study
DY382	MATalpha *ura3Δ0 his3Δ1 leu2Δ0 nab3Δ0*::*kanMX (lys2 and met15 status unknown)* [pRS316-*NAB3* (*URA3*)] [pRS315-nab3^24Q>E^ (*LEU2*)]	This study
DY384	MATalpha *ura3Δ0 his3Δ1 leu2Δ0 (lys2 and met15 status unknown) nab3Δ0*::*kanMX* [pRS315Nab3-14QE(*LEU2*)]	This study
DY3111	MATalpha *ura3Δ0 his3Δ1 leu2Δ0 (lys2 and met15 status unknown) nab3Δ0*::*kanMX* [pRS316-*NAB3* (*URA3*)]	This study
H-314	MATa *ura3-1 trp1Δ ade2-1 leu2-3*,*112 his3-11*,*15 pcf11Δ*::*TRP1* [pFL38-*PCF11* (*URA3*)]	Sadowski *et al* 2003

### Semi-denaturing detergent agarose electrophoresis

Purified protein samples were adjusted to 2% SDS, 140 mM β-mercaptoethanol, 10% glycerol, 0.002% bromphenol blue, 80 mM Tris, pH 6.8 and separated by electrophoresis in agarose gels (1.5% w/v) in 40 mM Tris-acetate, pH 7.8; 1 mM EDTA, 0.1% SDS run at 4°C. Bio-Rad Precision Plus Protein Kaleidoscope (Cat. No. 161–0375) molecular weight markers were run as standards. Proteins were blotted to Protran nitrocellulose transfer membrane (Whatman) by capillary action for 18 hrs. Filters were blocked in 5% (w/v) nonfat dry milk in Tris-buffered saline with 0.1% Tween-20 [29] and probed with rabbit anti-S tag antibody (catalog no. PM021; MBL International Corp., Woburn, MA).

### Electron microscopy

Five μl of sample suspension were placed on a 400-mesh carbon coated copper grid that had been made hydrophilic by glow discharge. After 5 minutes, the grid was rinsed by briefly touching the sample side to a drop of distilled water. The residual water was then removed by blotting to filter paper. For negative staining, 5μl 1% aqueous phosphotungstic acid (pH 6.5) was applied to the grid immediately after water removal, and excess liquid was removed by blotting after 30 seconds. The grid was air dried before viewing on a JEOL (Tokyo, Japan) IEM-1400 transmission electron microscope equipped with a Gatan (Pleasanton CA) 2k x 2k US1000 CCD camera. While filaments and less well-defined amorphous material were both apparent, the bulk of the negatively stained material resided in filaments. Representative images were captured digitally as.tif files.

### Thioflavin T fluorescence

Assays were carried out as described by LeVine [30]. Thioflavin T (Sigma Chemical, St. Louis, MO) was dissolved in 50mM glycine-NaOH, pH 8.5 and diluted to 10 μM when mixed with proteins. The fluorescence was read in a Shimadzu RF-5301PC spectrofluorophotometer in spectrum mode, with the excitation filter at 450+/-5 nm and the emission filter at 485+/-5 nm or using a Biotek Synergy 4 plate reader (384 well; 50 μl per well; triplicate wells) with excitation at 450 nm and emission at 490 nm.

## Results

We previously showed that a recombinant version of the *Saccharomyces cerevisiae* Nab3 LCD (its C-terminal 134 amino acids; “Nab3^134^”) formed amyloid polymers and an ordered hydrogel [6, 12]. This region of the protein is important for its function as a termination factor and for cell viability. To learn more about the sequence determinants of self-assembly, we examined Nab3^134^ with a specific set of mutations. Because we identified mutations in Nab3 that impact its termination activity by deleting all or part of 16 consecutive glutamines in the LCD [22, 23], we deleted this homopolymer tract (Fig 1A, ΔQ16). Filaments readily formed from this derivative as scored by electron microscopy (Fig 1B, middle). We then introduced a deletion of the terminal 18 amino acids which have structural homology to an α-helix Δα) of human hnRNP-C that can self assemble into a tetrameric bundle, and which is also important for Nab3’s termination function [12, 23] (Fig 1A, Δα). This purified protein was also competent to form filaments (Fig 1B, right panel). Although both of these segments are important for termination activity, neither the loss of the stretch 16 glutamines nor the terminal 18 residues were lethal in the context of the rest of the Nab3 protein [22].

Nab3^134^ is 32% glutamine, and it resembles prion-like domains of yeast that polymerize into filaments by stacking their β-sheet-forming, low complexity domains perpendicular to the direction of filament polymerization [31]. The primary sequence of domains such as these is less important than the overall amino acid composition, and the polymer fails to form if charged residues are introduced by mutation since they occur in-register in the β sheet stack providing unfavorable charge-repulsion [31]. To test if this was true for the Nab3 LCD, we substituted glutamate for glutamine at every-other position of runs of consecutive glutamines, as well as for isolated residues in the domain (Fig 1A). This change of 24 glutamines to glutamates altered the composition from 32 to 14% glutamine. This low-glutamine version of the recombinant Nab3 LCD was purified from *E*. *coli*. The introduction of charged glutamic acid residues resulted in an anomalously slow mobility of the polypeptide on SDS-PAGE relative to the wildtype LCD (Fig 1C) [6]. When subjected to filament forming conditions for the same length of time as that described for the wildtype Nab3^134^ domain, the low-glutamine Nab3 LCD failed to form filaments or a hydrogel, or to bind the β-sheet-binding dye thioflavin T (Fig 1D). Continued incubation for many months under filament forming conditions did not result in detectable polymer formation.

**Fig 2:**
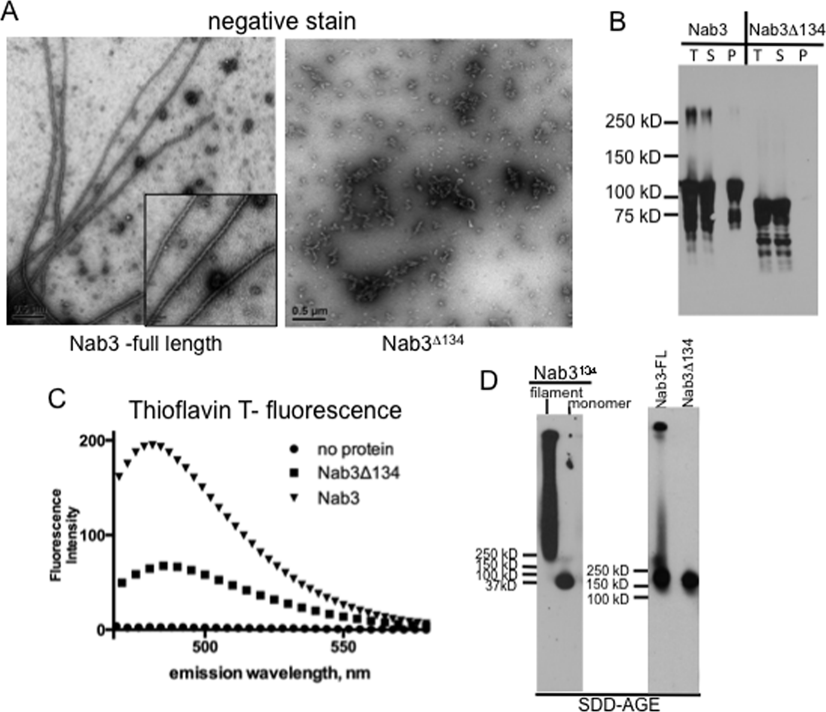
Amyloid formation by full-length Nab3 protein. Purified Nab3 and Nab3Δ134 were examined by transmission electron microscopy (A), or pelleted and subjected to Western blotting (B). Total (T), supernatant (S), and pellet (P) fractions were analyzed in (B). Nab3 and Nab3Δ134 were incubated with thioflavin T and examined by spectrofluorimetry at the indicated wavelengths (C) or subjected to SDD-AGE using anti-Nab3 antibody (D; right panel). As a control, Nab3^134^ filaments and monomers were analyzed on a separate gel with antibody against S-tag (left panel) by SDD-AGE.

Nab3^134^ represents the C-terminal 17% of the entire Nab3 protein. To test if it potentiates polymerization for full-length Nab3, we purified the entire 802-residue protein from *E*. *coli* with and without its final 134 amino acids and subjected the respective proteins to polymerization conditions. Full-length Nab3 formed filaments as scored by electron microscopy (Fig 2A). Assembly was dependent upon the C-terminal domain that autonomously forms filaments, since Nab3 lacking that region (Nab3Δ134) failed to show filaments (Fig 2A). Since the C-terminus of Nab3 is intrinsically unstructured and sensitive to proteolysis [12], we needed to show that the filaments were composed of full-length Nab3 and not the LCD released from the protein by spurious proteolysis following expression in *E*. *coli*. Filaments were pelleted by centrifugation (13,000 x g; 5 min) and analyzed by Western blotting with an anti-Nab3 antibody. We found that the pelletable fibers were indeed formed from full-length protein (Fig 2B; “Nab3” lane “P”). This procedure also confirmed that purified Nab3Δ134 did not assemble into filaments (Fig 2B, “Nab3Δ134” lane “P”). Thioflavin T-binding and fluorescence also revealed amyloid formation by the full-length protein (Fig 2C, ▼). Nab3 lacking the filament-forming C-terminal 134 amino acids bound the dye less well (■); residual fluorescence could be due to the glutamine/proline-rich bias of a small part of the prion-like domain still retained in this derivative [5].

An additional test for amyloid formation is the use of semi-denaturing agarose gel electrophoresis (SDD-AGE) to separate monomeric protein from high molecular weight polymers [32]. As shown previously, Nab3^134^ assembled into an SDS-resistant form that was readily separable from the soluble form of the protein (Fig 2D, left panel). Similarly, full-length Nab3 formed high molecular weight, SDS-resistant aggregates while Nab3 lacking the Nab3^134^ assembly domain did not (Fig 2D, right panel).

**Fig 3:**
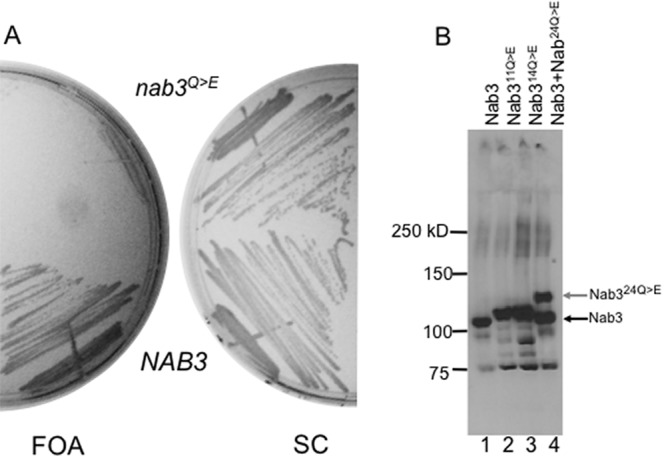
Nab3 whose LCD lacks 24 glutamines cannot support cell viability. (A) Yeast bearing a *URA3*-marked plasmid containing *NAB3* and either a *LEU2*-marked plasmid encoding wildtype *NAB3* (DY357) or one encoding *nab3* with the twenty-four glutamine to glutamate substitutions shown in Fig 1 (*nab3*^*Q>E*^) (DY382), were grown in SC leu^-^ and plated onto media containing FOA or synthetic complete (SC) media. (B) An anti-Nab3 western blot is shown on lysates from yeast strains DY357, DY368, DY384, and DY382 (lanes 1–4, respectively) containing the indicated versions of Nab3. (Control strains harboring as their only copy of *NAB3* either an 11 or 14 glutamine to glutamate substitutions are shown in lanes 2 and 3).

If assembly of Nab3 through its LCD is important for its function, the 24 glutamine to glutamate substitutions in the LCD that impair filament and hydrogel formation should impact cell physiology. To test this, we introduced this low-glutamine version of the LCD into full length Nab3 expressed from its own promoter and tested if it could support cell viability by plasmid shuffling. This test revealed that cells are not viable with only the mutant form of Nab3 (Fig 3A). To insure that the mutant Nab3 protein was synthesized, we examined cells with a plasmid expressing wildtype Nab3 and with one expressing the low-glutamine version of Nab3. We anticipated that full-length Nab3 with the 24 glutamine to glutamate substitutions (Fig 1A) would also display an unusually slow mobility on SDS-PAGE as observed above for the isolated domain (Fig 1C), and thus we would be able to distinguish the wildtype from the mutant protein in cells expressing both. This was indeed the case (Fig 3B, lane 4), and the mobility anomaly was further confirmed by comparing the 24 glutamine to glutamate version of Nab3 to derivatives of the protein with 11 or 14 glutamine to glutamate substitutions which expressed intermediate mobility on SDS-PAGE and could serve as the sole Nab3 in these strains (Fig 3B). Thus, Nab3^24Q>E^ was ineffective in supporting cell survival. In fact, when cells with both plasmids (one containing wildtype *NAB3* marked with *URA3* and another with Nab3^24Q>E^ marked with *LEU2*) were grown on media selective only for the latter (leucine-dropout media), both were retained because, even though there was no selection for *URA3*, wildtype *NAB3* had to be retained to cover for the inactive *nab3*^*24Q>E*^ allele.

**Fig 4:**
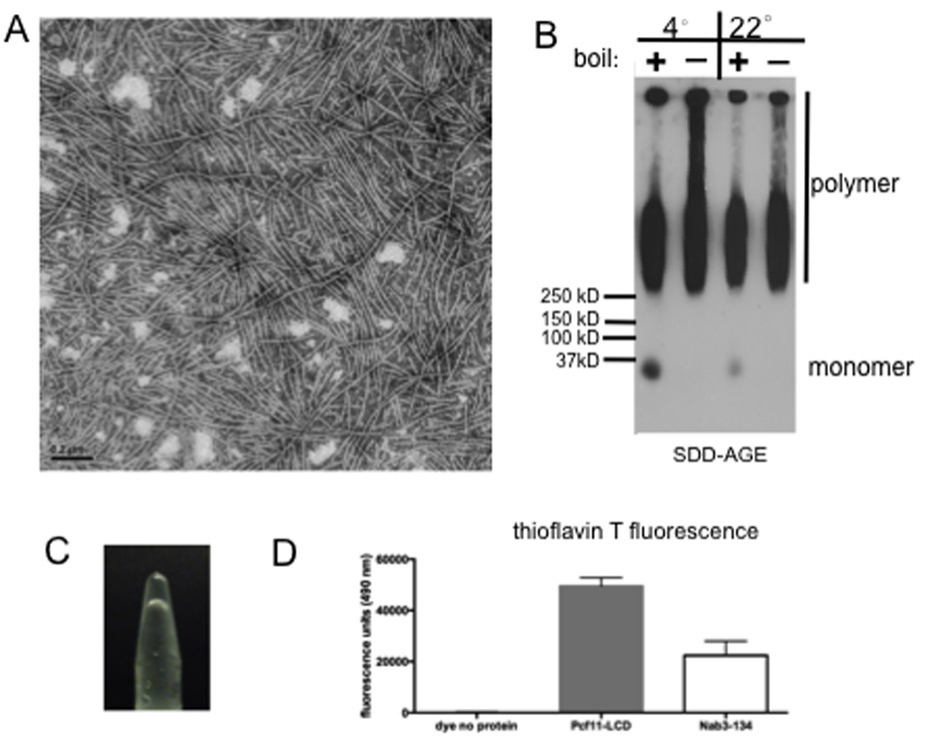
Pcf11’s LCD forms amyloid filaments and a hydrogel. (A) Transmission electron microscopy of a sample of purified Pcf11 LCD filaments. (B) Pcf11 protein incubated for filament formation at 4°C or 22°C was subjected to SDD-AGE and probed with anti-S-tag antibody. Samples were boiled (+) or incubated at 22°C (-) with SDS before loading as indicated. (C) Photograph of inverted tube holding a hydrogel formed from Pcf11’s LCD. (D) Average (n = 3) thioflavin T fluorescence at 490 nm of Pcf11’s LCD (20 μg) as compared to that of Nab3^134^ (30 μg). Error bars represent the standard deviation.

**Fig 5:**
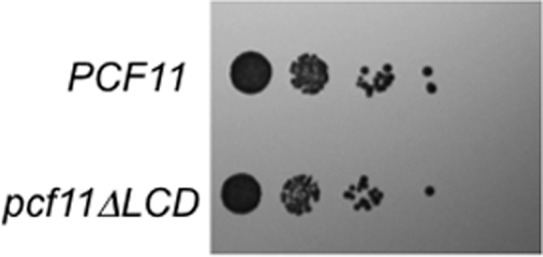
Pcf11’s LCD is not essential for viability. Yeast bearing a *LEU2*-marked plasmid with wildtype *PCF11* or *PCF11* from which the region encoding its LCD was deleted (*pcf11ΔLCD*), were grown in liquid media, serially diluted, spotted onto solid media, and grown at 30°C.

A number of yeast transcription termination factors have a low complexity domain that appears prion-like when analyzed by a Hidden Markov Model algorithm trained on well-characterized prions [5]. Since, we observed that the LCDs of Nab3 and Nrd1 both formed homopolymeric filaments, we tested if that from Pcf11 could as well. The low complexity domain purified from *E*. *coli* formed filaments that could be observed by electron microscopy (Fig 4A). Polymers could be formed at 4 or 22°C and they were resistant to boiling in the presence of SDS as seen by SDD-AGE analysis (Fig 4B). The filaments readily organized into a hydrogel (Fig 4C) and they bound thioflavin T and fluoresced in a manner similar to Nab3^134^ (Fig 4D). Deletion of the LCD of Nab3 compromises cell viability [9, 12]. To test if this is the case for Pcf11, a version of the protein lacking its LCD (residues 228–287) on a *LEU2*-marked plasmid was transformed into yeast cells harboring a *URA3*-marked plasmid encoding wildtype Pcf11. Cells that lost the latter plasmid were selected on FOA-containing medium and were able to grow well when the only copy of *PCF11* was the one lacking its LCD (Fig 5). Thus, Pcf11’s LCD is not required for cell viability.

## Discussion

**Fig 6:**
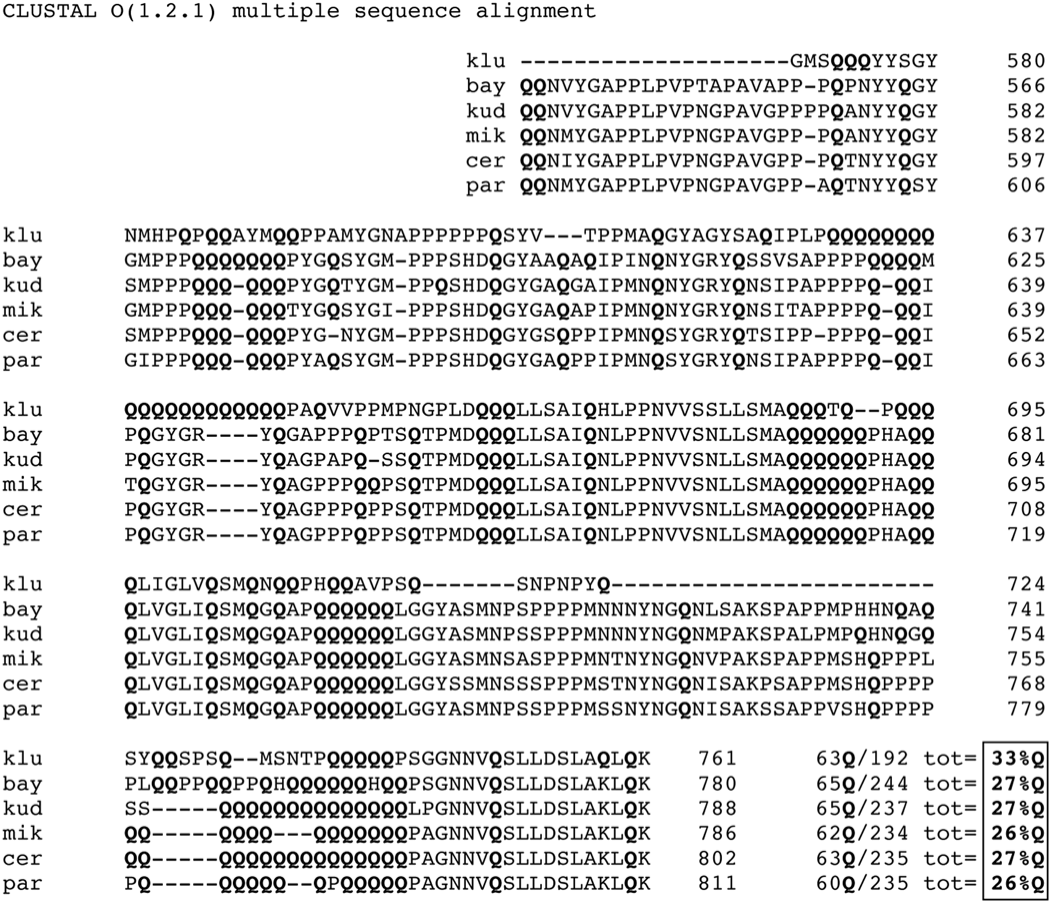
Alignment of *Saccharomyces* Nab3 LCDs. The prion-like domain of Nab3 as defined by Alberti *et al*. (2009) was aligned using CLUSTAL Omega with the cognate region from *S*. *kluyveri*, *S*. *bayanus*, *S*. *kudriavzevii*, *S*. *mikatae*, and *S*. *paradoxus*. The numbering of amino acids for each is shown at right. Glutamine residues (**Q**) were tallied for each species, divided by the total number of residues in its LCD (tot), and described as a percentage thereof in the box at lower right.

A growing body of evidence indicates that RNA binding proteins often possess low complexity, unstructured regions that can form amyloid polymers [2, 3, 33]. These domains are functionally important and are involved in the assembly of granules or droplets in living cells [3, 24, 26, 33, 34]. Here we provide evidence that the determinants of the Nab3 LCD’s ability to polymerize reside in the glutamine-rich quality of the domain. This is similar to what has been seen for the LCDs that enable prions to form filaments and render them prone to aggregation [35, 36]. When the LCD’s glutamine content is drastically reduced by mutation and introduced into the context of the full-length yeast Nab3 protein, it failed to support cell viability, suggesting that polymer-formation is important for its function. It remains possible that the increased charge introduced into Nab3 following the substitution of glutamines with glutamates, renders the protein non-functional for reasons other than its amyloid-forming capacity. In any case, genetic and biochemical evidence indicate that multiple copies of Nab3, with its partner Nrd1, associate with a nascent transcript to provoke transcription termination [9, 12, 22]. Also apparent, is the evolutionary plasticity of the LCD’s primary sequence, and particularly the glutamine positioning, as seen by its polymorphic nature which is apparent when *S*. *cerevisiae* Nab3 is aligned with that from five other *Saccharomyces* species (Fig 6). While the exact positions of the glutamines vary, the fractional glutamine composition has been retained in the range of 26 to 33% (Fig 6, box lower right), reinforcing the notion that the primary sequence is less important than overall composition [35, 36]. Interestingly, the very C-terminal region of Nab3 that has structural homology to an α-helical region of hnRNP-C and is not required for filament formation (Fig 1A), is amongst the best conserved parts of the LCD (Fig 6). This portion of Nab3 can self-assemble independently of the LCD, as can the cognate region of hnRNP-C [22, 37]. The domain is important for the termination function of Nab3 as revealed by mutations, including some that are structurally subtle such as a substitution of alanine for leucine only two positions from the C-terminus which reduces termination efficiency *in vivo* [22].

We also show that the Nab3 LCD, which independently forms amyloid polymers, is responsible for filament formation of the full-length protein with its RNA-binding domain. The finding that full length Nab3 forms filaments is an important extension of the initial computational analysis identifying LCDs in the yeast proteome that have assembly potential [5]. In this regard, we also show here that another prion-like domain identified in that study can assemble into filaments and a hydrogel *in vitro*. This domain is contained in the termination factor Pcf11, thereby extending the trend for termination complexes to contain subunits with this ability. Pcf11 co-exists in the CFI polyadenylation complex with Hrp1, whose prion-like domain scores very strongly in the study of Alberti *et al*., including the ability to form filaments *in vitro* [5]. Combined with the potential of LCD-containing Mediator subunits to polymerize [16], and the finding that fibrous polymers formed from LCDs interact with pol II’s CTD [38], LCD polymerization is a recurring feature of complexes that interact with pol II across the transcription cycle. It is also significant to note that we have found conditions in which the low complexity domains from three different proteins, Nab3, Nrd1, and Pcf11, form amyloid polymers. These were initially identified in the yeast proteome computationally but *in vitro* assembly had not been detected [5]. One possibility is that purification here was done in the absence of denaturants. Another consideration may be our use of thioredoxin as a fusion partner during expression in *E*. *coli*. In any case, our findings suggest that under certain conditions there may be additional members of the computationally-derived set of predicted yeast prion-like domains identified by Alberti *et al*. that indeed polymerize.
